# Forward Field Computation with OpenMEEG

**DOI:** 10.1155/2011/923703

**Published:** 2011-03-14

**Authors:** Alexandre Gramfort, Théodore Papadopoulo, Emmanuel Olivi, Maureen Clerc

**Affiliations:** ^1^Parietal Project Team, INRIA Saclay Ile-de-France, Neurospin-CEA, Bât 145, Point Courrier 156, 91191 Gif/Yvette, France; ^2^Athena Project Team, INRIA Sophia Antipolis-Méditerranée, 2004, Route des Lucioles, 06902 Sophia Antipolis, France

## Abstract

To recover the sources giving rise to electro- and magnetoencephalography in individual measurements, realistic physiological modeling is required, and accurate numerical solutions must be computed. We present OpenMEEG, which solves the electromagnetic forward problem in the quasistatic regime, for head models with piecewise constant conductivity. The core of OpenMEEG consists of the symmetric Boundary Element Method, which is based on an extended Green Representation theorem. OpenMEEG is able to provide lead fields for four different electromagnetic forward problems: Electroencephalography (EEG), Magnetoencephalography (MEG), Electrical Impedance Tomography (EIT), and intracranial electric potentials (IPs). OpenMEEG is open source and multiplatform. It can be used from Python and Matlab in conjunction with toolboxes that solve the inverse problem; its integration within FieldTrip is operational since release 2.0.

## 1. Introduction

It is well recognized that conductivity models and forward solutions play an important role in accurate source localization from EEG [1, 2]. This is also true for MEG, though to a lesser degree [3].

Despite the simple mathematical nature of the equations giving rise to the electric potential and the magnetic field, these equations are not trivial to solve numerically, because of large conductivity ratios arising between neighboring tissues of the head. The field of forward modeling in EEG and MEG dates back to Barnard, who derived integral equations for electrocardiography [4, 5] and to Geselowitz [6]. After these seminal papers, several groups proposed Boundary Element solutions to these problems (as well as other types of solutions, notably Finite Elements, that are beyond the scope of this paper) [7–9].

The difficulty in the numerical resolution of the forward problem arises when electric sources are close to the boundary between two such tissues, in which case the solvers face accuracy problems [8]. Such source positions are not rare occurrences: indeed the gray matter, where the electric dipoles representing brain activity may be assumed to reside, is quite close to the cerebrospinal fluid (CSF) and to the skull. An accuracy correction method, called Isolated Skull Approach (ISA), was proposed to alleviate these accuracy issues [10]. Although the accuracy was improved in most cases, it was sometimes degraded [8]. Until recently, no acceptable solution was available that did not use the ISA.

For these reasons, a research program on forward modeling for EEG and MEG was conducted at INRIA, leading to the development of the symmetric BEM [11–13]. The OpenMEEG software package makes this new development available to the MEG/EEG community.

The thrust of OpenMEEG is to propose accurate forward problems, in several instances. The most classical instances are EEG and MEG, but OpenMEEG also allows to compute the electric potential due to boundary current injection (as occurs in Electrical Impedance Tomography or in Functional Electrical Stimulation) and to compute the electric potential measured within the brain (as occurs in stereographic EEG).

For each of these instances, the result of the forward problem is expressed as a lead field, that is, the matrix representing the linear relation between sources and measurements, a.k.a. “Gain matrix.”

The modeling assumptions of OpenMEEG are explained in Section 2.Section 3 then details the four instances of forward computation: EEG, MEG, EIT, and Internal Potential (IP), from the physical model to the OpenMEEG commands.  Section 4 provides practical information on OpenMEEG usage. The accuracy of OpenMEEG is assessed in a benchmark comparison test in Section 5, and the paper ends with a conclusion. The material presented here refers to releases 2.1 and later.

## 2. Modeling Assumptions

The quasistatic regime of Maxwell's equations is valid at the frequencies of interest in EEG and MEG, and also for EIT and functional electrical stimulation, at stimulation frequencies below 1 kHz. In this regime, the electrical potential *V* is governed by the law of electrostatics

(1) $$\nabla \cdot \left(\sigma \nabla V\right)=\nabla \cdot J^{p},$$

where *σ* is the conductivity field and **J** ^**p**^ is the source distribution. The brain sources are modeled as dipoles, representing average postsynaptic currents within pyramidal cortical neurons. A boundary condition fixes the value of the normal current on the domain boundary

(2) σ∇V·n=j.

In EEG and MEG, the value of the normal current on the scalp is *j* = 0, but in electrical impedance tomography, *j* takes the values of the current injected on the scalp.

### 2.1. Head Model

**Figure 1:**
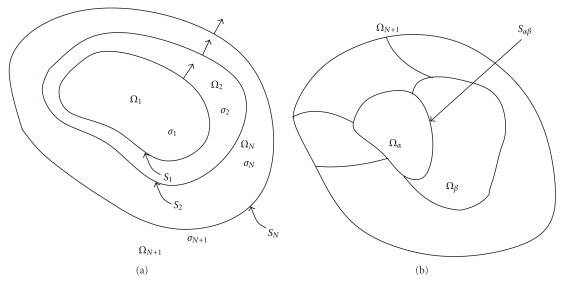
Boundary Elements are well suited for piecewise constant isotropic conductivity models. OpenMEEG handles nested regions (a) and could in principle be extended to more general, disjoint regions (b) [13].

OpenMEEG is based on a Boundary Element representation of physical fields, implying that the conductivity model, describing the conductivity field *σ*, must be piecewise constant. The physical fields are thus represented on the boundaries of the regions of constant conductivity. More precisely, OpenMEEG is restricted to nested conductivity models, that is, in which there are successive layers of constant conductivity (see Figure 1(a)). This model is generally well suited to the head, as it can handle the brain, CSF, skull, and scalp conductivities. Extensions of the symmetric BEM have been proposed to handle nonnested regions as in Figure 1(b) but are not yet handled by OpenMEEG [13]. Regarding the conductivity field, the only theoretical restriction for using Boundary Element methods is that the conductivity field must be translation invariant in each domain. Thus, for complex 3D domains as the head, anisotropic conductivity cannot be handled with a BEM, and other solvers using volumic approaches must be used (e.g., Finite Element methods).

### 2.2. Source Models

 The primary current within the brain **J** ^**p**^ in (1) is represented as a distribution of dipoles. This distribution may be either pointwise or surfacic. A pointwise source distribution is a collection of pointwise dipoles, defined by their positions and moments. A surfacic source distribution is defined over a surfacic mesh, as

(3) $$J^{p}\left(r\right)=\underset{i}{\sum }\phi_{i}\left(r\right)J_{i}n\left(r\right),$$

where the sum runs over all vertices, *ϕ*_*i*_ is the piecewise linear function equal to 1 on vertex *i* and 0 on all others, and **n**(*r*) is the normal to the surface at position *r*. The source intensity is linear on each triangle and equal to *J*_*i*_ on vertex *i*. 

Note that the pointwise source distribution is the most commonly used, because it is difficult to define a surface supporting the sources—hence matching the gyri and sulci—on which the orientations are sufficiently smooth.

Another type of source that can be considered is the normal component of the boundary current: *j* in (2). This normal current is modeled as piecewise constant on the mesh, that is,

(4) $$j\left(r\right)=\underset{k}{\sum }\psi_{k}\left(r\right)j_{k},$$

where the sum here runs over all triangles and *ψ*_*k*_ is the piecewise constant function equal to 1 on triangle *k* and 0 on all others.

### 2.3. Sensor Models

 Four types of modalities are considered: EEG electrodes, MEG sensors, current injection electrodes, and intracranial electrodes for measuring the potential. In each case, the sensor model considered by OpenMEEG is very basic, that is, it does not model capacitive effects, nor electrode extension.

EEG and intracranial electrodes are assumed punctual and defined by their 3D coordinates. In the case of EEG, the 3D electrode position is projected orthogonally onto the scalp surface. Each MEG sensor is defined by a collection of points and weights, thus modeling magneto- or gradiometers, possibly with the shape of the coil wiring. Current injection electrodes are defined by their 3D coordinates, and the current injection model is a uniform current over the closest triangle to the injection electrode.

## 3. Forward Field Computation

For the models explained above, OpenMEEG is equipped to compute four different types of lead fields. We now detail the computations for each of them. In addition, the reader can refer to a global flowchart in Figure 5, which explains the structure of the commands and of the input/output arguments. Information on input/output format is provided in Section 4.

### 3.1. EEG Lead Field

Computing an EEG lead field amounts to computing the potential *V* on electrodes, due to a set of dipolar sources at prescribed positions and orientations. (For simplicity, our description considers a pointwise source distribution, but the method also applies to a surfacic source distribution) The potential *V* is defined, up to an additive constant, as the solution of (1) with a boundary condition (2) in which no current flows across the scalp. Considering a nested conductivity model as in Figure 1(a), the symmetric Boundary Element expresses the solution of this problem, restricted to the domain boundaries, as the solution of the set of equations

(5) $$HeadMatrix\cdot \left[\begin{array}{c} V_{S_{1}}\\ {\left(\sigma \partial_{n}V\right)}_{S_{1}}\\ V_{S_{2}}\\ {\left(\sigma \partial_{n}V\right)}_{S_{2}}\\ \vdots \\ V_{S_{N}} \end{array}\right]=SourceMatrix\cdot \left[\begin{array}{c} J_{1}\\ J_{2}\\ \vdots \\ J_{p} \end{array}\right]$$

for a set of *p* source intensities corresponding to *p* prescribed dipoles [11]. Both the potential *V*_*S*_*i*__ and the normal current (*σ*∂_**n**_ *V*)_*S*_*i*__ are discretized on each boundary *S*_*i*_ (except the scalp where only the potential needs to be discretized since the normal current vanishes). The potential is represented with piecewise linear boundary elements, while the normal current is represented with piecewise constant boundary elements.

The two matrices HeadMatrix and SourceMatrix involve Boundary Integral operators which OpenMEEG is equipped to compute. Computing the EEG lead field *L*_EEG_ amounts to solving the symmetric linear system (The denomination “Symmetric BEM” is due to the symmetric nature of the HeadMatrix):

(6) HeadMatrix·X=SourceMatrix

and applying to the result *X* an interpolation operator to infer the potential at the scalp electrode positions

(7) $$L_{\text{EEG}}=Head2EEGMatrix\cdot X.$$

Matrices are assembled in OpenMEEG by invoking the command

(8) om_assemble  Option  Parameters  Matrix.

HeadMatrix is assembled with the -HeadMat option and Parameters containing the geometry and conductivity description.

Head2EEGMatrix is assembled with the -Head2EEGMat option, and the same Parameters.

SourceMatrix is assembled with the -DipSourceMat or -SurfSourceMat option, depending on the source model (Section 2.2), and Parameters containing the geometry, conductivity, and source description (positions and orientations, or surface supporting a surfacic source).

Finally, (6) and (7) are solved by successively

- inverting matrix HeadMatrix:
  (9) om_minverser  HeadMatrix  HeadMatrixInv;
- applying the interpolation and the inverse matrix to the SourceMatrix:
  (10) $$\begin{array}{cc} & om\_gain\;\;\text{-}EEG\;HeadMatrixInv\\ & SourceMatrix\;Head2EEGMatrix\;GainMatrix. \end{array}$$

The EEG lead field is the output of the previous command.

### 3.2. MEG Lead Field

The magnetic field **B** depends both on the electric potential *V* and on the current source distribution **J** ^**p**^, through the Biot and Savart law

(11) $$B\left(r\right)=\frac{\mu_{0}}{4\pi }\int \left(J^{p}\left(r^{\prime }\right)-\sigma \nabla V\left(r^{\prime }\right)\right)\times \frac{r-r^{\prime }}{{||r-r^{\prime }||}^{3}}dr^{\prime },$$

when *j* = 0 on the boundary.

The magnetic field **B** can be split into two contributions, the primary field generated by the primary current and the ohmic field. The primary field is computed as a linear relation between sources and measurements, via a matrix Source2MEGMatrix. The Ohmic field is computed as a linear relation between electrical potential and measurements. Computing this Ohmic lead field amounts to solving (6) (as when computing *L*_EEG_) and applying to the result *X* an operator Head2MEGMatrix. Finally the MEG lead field *L*_MEG_ is equal to:

(12) $$\begin{array}{cc} L_{\text{MEG}} & =Head2MEGMatrix\cdot X+Source2MEGMatrix. \end{array}$$

HeadMatrix and SourceMatrix are identical to those of the EEG* lead field,* and their assembly has been explained in Section 3.1. Matrices Head2MEGMatrix and Source2MEGMatrix are obtained through the om_assemble command. Head2MEGMatrix is assembled with the option -Head2MEGMat and with Parameters describing the geometry, conductivity, and sensors; Source2MEGMatrix is assembled with the option -DipSource2MEGMat (pointwise source) or -SurfSource2MEGMat (surfacic source), and with the previously listed parameters, plus the source description (discrete points and orientations, or a surface). Finally, the MEG lead field *L*_MEG_ is computed by invoking om_gain with the option -MEG, and input matrices HeadMatrixInv, SourceMatrix, Head2MEGMatrix, Source2MEGMatrix.

**Figure 2:**
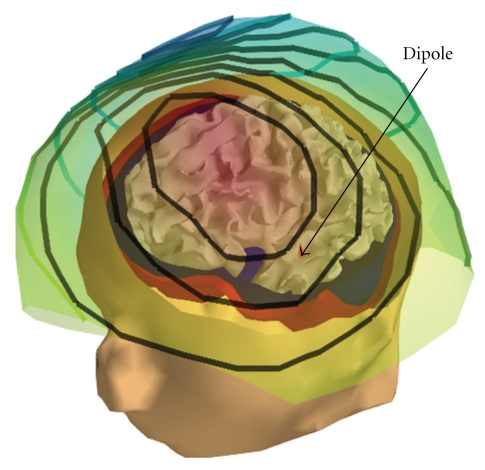
MEG simulation: visualization on a surface interpolating the sensors (radial magnetometers) of the field induced by a dipolar source on the left temporal cortex (in red). Computation is done with OpenMEEG and a 3-layer head model.

Figure 2 displays a magnetic field corresponding to a single dipole and interpolated on a surface containing the magnetometer positions.

### 3.3. EIT Lead Field

OpenMEEG also allows to compute the electric potential due to an applied current on the boundary of the domain. This occurs in electrical impedance tomography, and also in functional electrical stimulation. We will denote this type of problem “EIT,” bearing in mind that it may also concern other application fields. Electrical Impedance Tomography (EIT) seeks to estimate the conductivities of the model, by analyzing the potential resulting from the application of a current on the boundary. In EIT, the conductivities must then be adjusted to match the measured current potential correspondence [14–16]. OpenMEEG allows to compute this current potential correspondence, for fixed values of conductivity. This amounts to solving (1) and (2), for prescribed injected current *j*, and selecting the values of the potential on the electrodes as for EEG in (7).

It is interesting to note that only the right-hand side of (6) must be changed when EIT is being solved instead of EEG. The source matrix for EIT is computed by invoking om_assemble with the -EITSourceMat option and as parameters the geometry file, conductivity file, and the file describing the EIT electrodes.

After inverting the left-hand side matrix in (6) (yielding HeadMatrixInv) and computing the electrode interpolation matrix Head2EEGMatrix, the EIT lead field is computed using om_gain with the -EEG option

(13) $$\begin{array}{cc} & om\_gain\;\text{-}EEG\;HeadMatrixInv\;\;EITSourceMatrix\\ & Head2EEGMatrix\;GainMatrix. \end{array}$$

**Figure 3:**
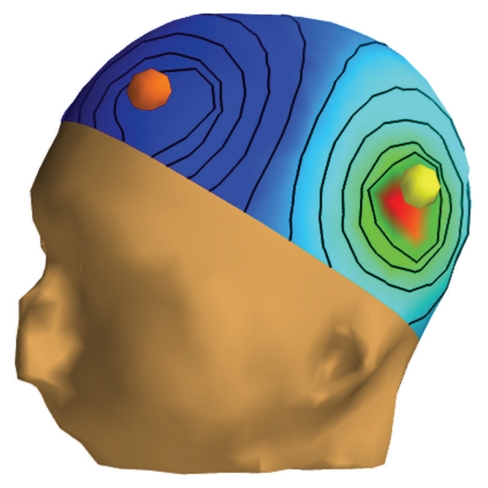
EIT simulation: visualization on a surface interpolating EEG electrodes of the electric potential when a current flows from one electrode (in yellow) to another (in orange). Computation is done with OpenMEEG and a 3-layer head model.

Figure 3 displays the scalp potential corresponding to a current injection between two electrodes.

Note that, for a new set of conductivity values, the computation of the HeadMatrix is immediate, because of the form of the HeadMatrix (14) (refer to [11] for a proof). This makes the EIT inverse problem quite tractable using OpenMEEG [17]:

(14) $$HeadMatrix=\overset{N}{\underset{i=1}{\sum }}\left(\sigma_{i}A_{i}+\sigma_{i}^{-1}B_{i}\right).$$

### 3.4. Internal Potential (IP) Lead Field

 In certain clinical settings, the potential may be measured within the brain (intracranial EEG or stereotaxic EEG). Given a distribution of current generators within the head, OpenMEEG is able to compute the potential at any position within the head (brain, skull, scalp). This may appear surprising, because OpenMEEG is based on a Boundary Element Method that, by definition, only represents the potential on the interfaces between domains. But computing the potential within a domain from the knowledge of the potential and normal current on the surrounding interfaces is only a matter of applying a Green harmonic representation theorem.

In practise, this relation is provided by a matrix Head2IPMatrix. One must also take into account a contribution from the sources belonging to the same domain as the electrodes.

Computing the internal potential lead field *L*_IP_ proceeds by solving for *X*, as for the computation of *L*_EEG_,

(15) HeadMatrix.  X=SourceMatrix,

and then computing

(16) $$\begin{array}{cc} L_{\text{IP}} & =Head2IPMatrix\cdot X+Source2IPMatrix. \end{array}$$

Head2IPMatrix is assembled with the om_assemble command with option -Head2InternalPotMat and the usual parameters (geometry and conductivity description) along with the internal points. Source2IPMatrix is assembled with the same command with option -DipSource2InternalPotMat and, in addition to the previous parameters, the source description. Finally, *L*_IP_ is computed by invoking om_gain with the option -InternalPotential and input matrices HeadMatrixInv, SourceMatrix, Head2IPMatrix, and Source2IPMatrix.

**Figure 4:**
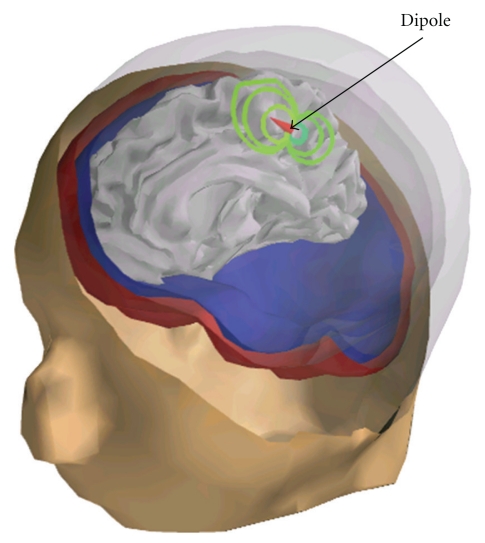
Internal Potential simulation: visualization of the internal potential computed using OpenMEEG in a 3-layer head model. A dipolar source (red cone) is located in the left hemisphere of the cortex (not represented), and the curved lines are isopotential lines. A bending of the isopotentials near the skull can be observed.

Figure 4 displays the internal potential due to a single dipole. 

## 4. Usage of OpenMEEG

### 4.1. I/O File Formats

OpenMEEG handles several file formats corresponding to several types of objects: vectors, matrices, head geometries, meshes, dipoles, conductivities, and sensors.

By default, matrices and vectors are stored on disk using a Matlab file format. Symmetric matrices, for which Matlab does not propose a format, are represented as a Matlab structure. Alternatively OpenMEEG handles plain ASCII files (usually used for sensors and dipole descriptions) and BrainVisa textures.

OpenMEEG geometrical models are described via several files. Note that OpenMEEG considers SI units (point coordinates should be expressed in meters (m), conductivities in S/m, etc.). The top level file (with the extension  .geom) describes the nested structure of the different domains (see Figure 6). An associated conductivity file (with extension .cond) contains the conductivities of the domains (see Figure 7).

Mesh formats supported are BrainVisa  .tri files (default) and ASA  .bnd files.

### 4.2. Example Scripts and Demos

**Figure 5:**
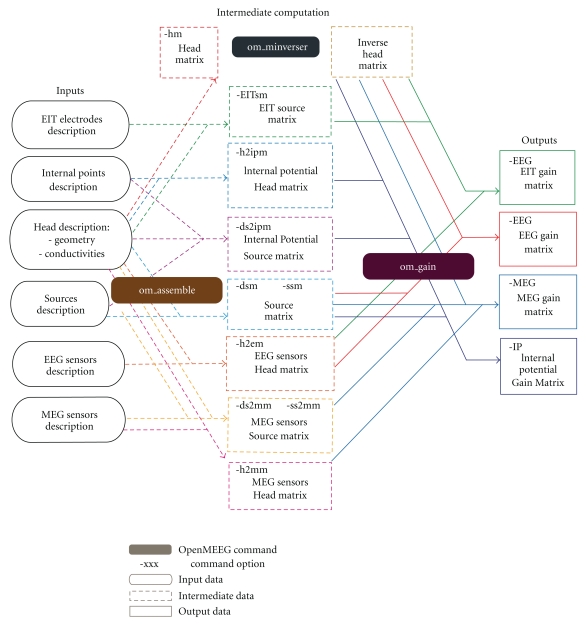
Flowchart describing the OpenMEEG procedure for computing EIT, EEG, MEG, and IP lead fields.

**Figure 6:**
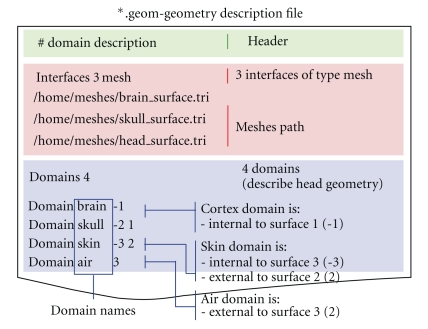
Sample geometry file. The Interfaces section provides the meshes, while the section Domains informs of the physical model.

**Figure 7:**
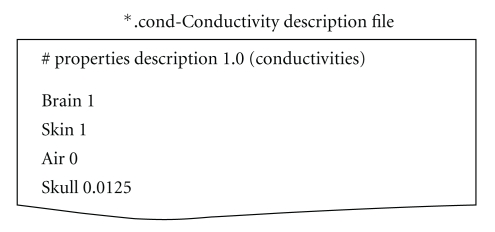
Sample conductivity file associated to the geometry file in Figure 6.

Much effort has been devoted to facilitating the use of OpenMEEG by the M/EEG community. OpenMEEG can be invoked either via a command line interface (see Figure 5) or via higher-level languages. OpenMEEG can be used from Python or from Matlab via the FieldTrip Toolbox, where it is fully integrated in the forward modeling routines. Within FieldTrip, OpenMEEG can compute lead fields for head models with 1, 2, 3, or 4 nested layers.

Algorithms 1 and 2 provide sample Python and FieldTrip scripts. 

### 4.3. Technical Details

 OpenMEEG is distributed under the French open source license CeCILL-B, intended to give users the freedom to modify and redistribute the software. It is therefore compatible with popular open source licenses such as the GPL and BSD licenses. Due to the CeCILL-B license, anybody distributing a software incorporating OpenMEEG has an obligation to give credits by citing the appropriate publications. (The references to be cited to comply with the license can be found on the OpenMEEG webpage http://openmeeg.gforge.inria.fr.)

OpenMEEG is implemented in C/C++ with limited external dependencies. It uses the Intel MKL libraries on Windows and ATLAS (BLAS/LAPACK) on Unix systems for fast and accurate linear algebra. A modified version of the MATIO library (http://sourceforge.net/projects/matio) has been integrated in OpenMEEG for Matlab compatibility. The source code of OpenMEEG is hosted on the INRIA GForge platform and is accessible from http://openmeeg.gforge.inria.fr. 

OpenMEEG is available as precompiled binaries for GNU-Linux systems, Mac OS, and Windows (32 and 64 bits). OpenMEEG's build and packaging system is based on *CMake/CPack* (http://www.cmake.org) allowing easy development and deployment on all architectures. 

**Figure 8:**
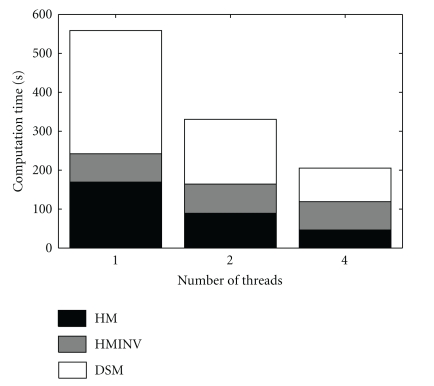
Computation time of an EEG lead field with the head model of Figure 4 (approximately 700 vertices per layer, 3 layers and 15000 dipoles). With 4 threads, the computation is almost 3 times faster. These results were obtained on a quad-core Intel Xeon CPU working at 3.20 GHz.

To accelerate the computations, OpenMEEG can be compiled with OpenMP, a technology that enables parallel computation at limited cost in terms of software design. The numerical integration, on which most of the computation time is spent, can then be run in parallel.  Figure 8 presents observed computation times for the computation of an EEG lead field with the head model of Figure 4 (approximately 700 vertices per layer, 3 layers and 15000 dipoles). It can be observed that with 4 threads, the computation is almost 3 times faster. The memory requirement for this example is 770 MB. The computation time of a forward field computation with OpenMEEG can be roughly broken down into three main components: the Head Matrix assembly, its inversion, and the Source Matrix assembly (identified as HM, HMINV, and DSM in Figure 8). 

 Deployment on multiple architectures with heterogenous hardware and software environments requires testing procedures to assess the stability of the solutions provided by compiled binaries. This testing procedure, based on *CMake/CTest*, guarantees the integrity of the results, in particular by comparison with analytical results on spherical models.

## 5. EEG and MEG Comparison Study

### 5.1. Benchmark Presentation

 When the head model consists of nested concentric spheres, the accuracy of EEG and MEG forward computations can be assessed by comparing the computed solution with the analytical solution. We here present an excerpt of the benchmark study presented in [18]. 

The precision of a forward solution is tested with two measures: the Relative Difference Measure (RDM) and the magnitude ratio (MAG) [19]. 

The RDM between the forward field given by a numerical solver *g*_*n*_ and the analytical solution *g*_*a*_ is defined as

(17) $$\text{RDM}\left(g_{n},g_{a}\right)=||\frac{g_{n}}{||g_{n}||}-\frac{g_{a}}{||g_{a}||}||\in \left[0,2\right],$$

while the MAG between the two forward fields is defined as

(18) $$\text{MAG}\left(g_{n},g_{a}\right)=\frac{||g_{n}||}{||g_{a}||}.$$

In both of these expressions, the norm is the Euclidian *ℓ* ^2^ norm over the set of sensor measurements.

**Geometrical Models**

The comparisons were made both on classic regular sphere meshes as in Figure 10, and on random meshes [18]. The BEM solvers are tested with three-layer head models which model the inner and outer skull, and the skin. The radii of the 3 layers are set to 88, 92, and 100, while the conductivities of the 3 homogeneous volumes are set to standard values: 1, 1/80 (skull) and 1. For every head model, solvers are tested with the same 5 dipoles positioned on the *z*-axis with orientation (1,0,1) and various distances to the inner layer (cf. Figure 10).

**Results: Accuracy of the Electric Potential Simulations**

Alternative BEM software available to the community are all based on the Geselowitz formulation [6]. From this formulation, different implementations may be derived. The potential may be modeled either with constant elements (i.e., the potential is piecewise constant over each mesh) or, for more precision, with linear elements (i.e., the potential is piecewise linear over each mesh). The computation may then be achieved with a Galerkin method involving numerical integration, as in OpenMEEG, or with a more simple collocation method (see [8] for a detailed study of Galerkin versus collocation methods). The linear collocation (LC) method is implemented by BEMCP [20] which is available from FieldTrip and is the default forward solver in SPM. In order to improve the accuracy of LC methods, the Isolated Skull Approach (ISA) has been proposed [10]. It is used by SimBio [21], Dipoli [22], and the Helsinki BEM [23], which implements both a simple LC and an LC with ISA (LCISA). Within SimBio, we only consider here its BEM solver, referred to as SimBio-BEM [24], as opposed to SimBio-FEM [25, 26] that focuses on inhomogeneous and anisotropic head volume conductor models.

The Helsinki BEM is the implementation used in this benchmark. However all the aforementioned solvers have been tested, and it has been confirmed that all LCISA solvers tested provide almost the same results, as do all the LC solvers tested [18]. One of the features of OpenMEEG is to use an adaptive numerical integration method. To demonstrate its influence on the results, we have also tested a nonadaptive version of OpenMEEG (OMNA).

For the sake of completeness, let us mention that the BEM solver implemented in the MNE (http://www.nmr.mgh.harvard.edu/martinos/userInfo/data/sofMNE.php) software package is also LCISA based.

**Figure 9:**
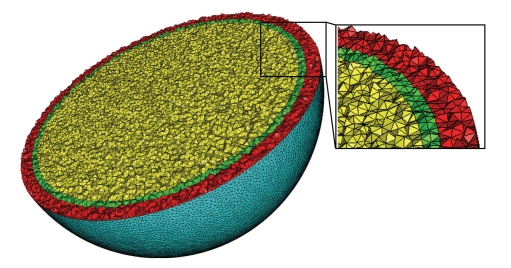
The 3D mesh used in FEM computations. The 3 layers are shown in red, green, and yellow, respectively.

**Figure 10:**
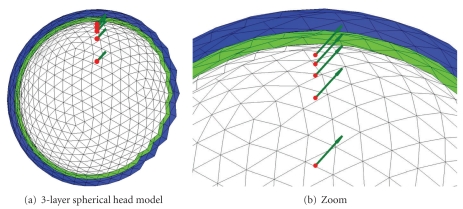
Head model made of 3 nested regularly meshed spheres with 5 dipoles close to the inner layer.

Furthermore, as a crude comparison, a basic finite element method with P1 basis elements on a tetrahedral mesh (TFEM) has also been run. It is a classical FEM, with a preconditioned conjugated gradient as solver (Jacobi preconditioner), and the dipole source is modeled through partial integration. Such a model approximates the dipole with a continuous distribution of sources supported over a small region, which introduces a source approximation error which does not exist for BEM models. Note that there are solutions to better model dipole sources with FEM such as the subtraction method or the Venant direct approach [27]. Such methods are beyond the scope of this contribution. The mesh (427,000 vertices, with 43,768 vertices on the outer surface) was generated with CGAL (CGAL, Computational Geometry Algorithms Library, http://www.cgal.org.) A view of this mesh is shown in Figure 9.

**Results: Accuracy of the Magnetic Field Simulations**

Magnetic fields are commonly computed, in the MEG community, using analytical solutions on spheres. While (Ohmic) volume currents do not contribute to the radial component of the magnetic field on a nested spherical model; they do on a realistic geometry and must then be computed. OpenMEEG and SimBio-BEM are two freely available software projects that provide a computation of the magnetic field depending on the electrical potential.

### 5.2. Results

#### 5.2.1. Regularly Meshed Spheres

**Figure 11:**
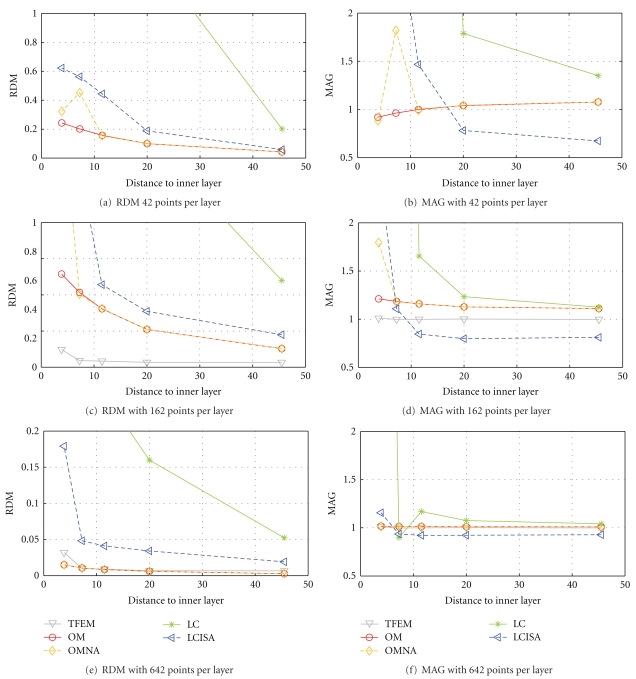
Forward EEG: accuracy comparison of different BEM solvers with three-layer sphere head models. We observe that the symmetric BEM outperforms the other BEM methods in term of precision. The TFEM was run on a mesh with 427,000 vertices, and the results were interpolated to 162 points in (c) and (d) and 642 points in (e) and (f).

 Results on regularly meshed spheres are presented in Figure 11, for 3 different point samplings on each interface. The coarsest sampling has only 42 vertices per layer and 42 EEG electrodes, the intermediate one has 162 points per layer and 162 EEG electrodes, and the finest sampling has 642 points per layer and 642 EEG electrodes. 

 From these simulations the following can be observed.

- The simple linear collocation method is clearly the least accurate.
- The linear collocation method with ISA correction is more accurate.
- OpenMEEG provides the most accurate solutions even when no adaptive integration is used. The adaptive integration further improves the results, particularly when the meshes are coarsely sampled (42 and 162 vertices per layer).
- Despite the high resolution of the mesh used with the FEM, OpenMEEG is more accurate for the model with 642 vertices per layer.

#### 5.2.2. Randomly Meshed Spheres

 Simulations have also been run on a large number of randomly meshed spherical meshes, in order to study the robustness of the solvers. Please refer to [18] for the meshing procedure. The results are obtained by testing each solver on 100 random head models. The mean accuracy measures (RDM and MAG) are represented using box plots.

**EEG Results**

**Figure 12:**
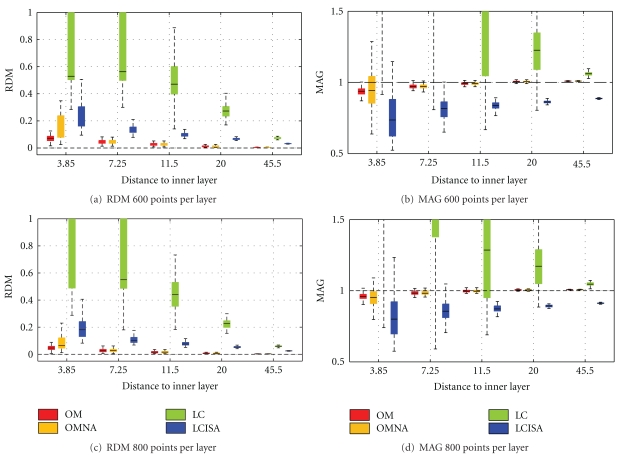
Forward EEG: RDM and MAG box plots obtained on 100 random 3-layer sphere models. Each layer contains 600 or 800 random vertices.

Figure 12 presents the box plots obtained by running the solvers on random head models with either 600 or 800 vertices per layer. The mean results follow the ranking of Figure 11. However the variances tell us that OM is not only very accurate, but also very precise because of its very small variance. (For the distinction between accuracy and precision, refer to http://en.wikipedia.org/wiki/Accuracy_and_precision.). The OMNA solver is also accurate but less precise: it has a larger variance, demonstrating that the adaptive integration improves robustness to the meshing. Linear collocation with ISA gives intermediate results. Also observe that linear collocation without ISA has significantly larger variance than the other solvers, meaning that it is very sensitive to the meshing.

**MEG Results**

**Figure 13:**
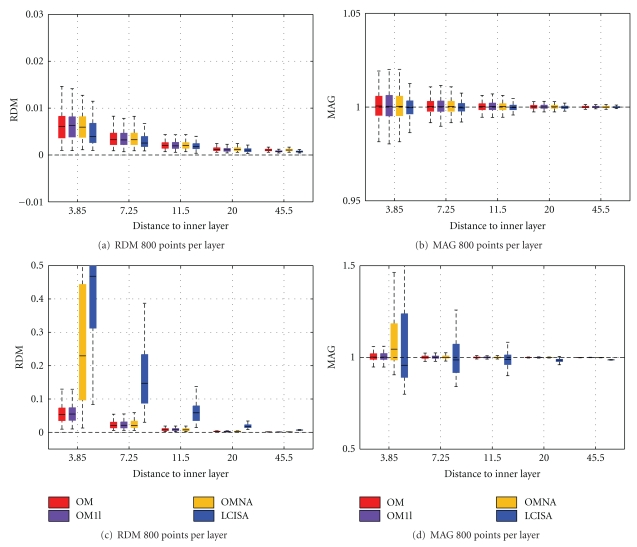
Forward MEG: RDM and MAG box plots obtained on 100 randomly meshed sphere models. OM and OMNA use a 3-layer model while OM1l and LCISA use a one-layer model. Each layer contains 800 random vertices. In (a) and (b), *radial magnetometers* are considered, whereas in (c) and (d) use *nonradial magnetometers*.

As explained in Section 3.2, the magnetic field depends on the electric potential, thus its accuracy follows from that of EEG. Although MEG machines generally only provide radially oriented sensors with respect to the helmet (except for some reference channels), we have, in the following experiments, computed the nonradial magnetic field in order to validate the Ohmic field contribution. Indeed, in spherical geometry, for radial sensors, the magnetic field does not depend on the Ohmic contribution—which is no longer true for more realistic head models. Two types of sensors were thus considered: a set of magnetometers oriented in the Cartesian direction (1,0,1) and located at a distance 120 from the center of the model and one set of radially oriented sensors at the same locations.  Figure 13 presents, for both types of sensors, the results of OpenMEEG on a 3-layer model, with and without adaptive integration (OM and OMNA), OpenMEEG on a one-layer model (OM1l), and LCISA (SimBio-BEM implementation) on a one-layer model. The use of a 3-layer model with OpenMEEG slightly improves the results obtained with only one layer. For radial magnetometers, one notices a slight advantage to LCISA both for accuracy and precision, but for nonradially oriented sensors, OpenMEEG outperforms both OMNA and LCISA. Performances of LCISA can however be significantly improved by increasing the number of vertices in each layer. In our investigations with SimBio-BEM, a number of 3400 nodes led to an RDM of maximally 0.047 and a MAG above 0.97.

## 6. Conclusion

 OpenMEEG is a comprehensive, open source software package for solving many different instances of forward problems in quasistatic electromagnetism. It can compute lead fields for EEG and MEG, as well as EIT (or Functional Electrical Stimulation) and intracerebral EEG. Regarding accuracy, OpenMEEG represents the state-of-the-art. Besides its excellent accuracy and its versatility, several other features of this software make it unique:

- the number of nested layers is unrestricted;
- dipolar sources may be positioned within any of the domains;
- EEG, EIT, MEG, and IP lead fields can be jointly computed on the same head model;
- it is interfaced with Python and Matlab via FieldTrip for a maximal ease of use.

The progress brought forth by this new software however only represents a limited contribution in modeling brain functional activity. Head model generation is a crucial problem in practice, and the need for automatized procedures in this domain is crying. When more complex head models (involving inhomogeneous and anisotropic conductivity) are needed, Boundary Element Methods are no longer applicable, and one must resort to Finite Element Methods, of which few open source solvers are yet available (SimBio-FEM [21]). Nevertheless, for the head models commonly used in practice, OpenMEEG represents the state of the art for forward computation.

## Figures and Tables

**Algorithm 1:**
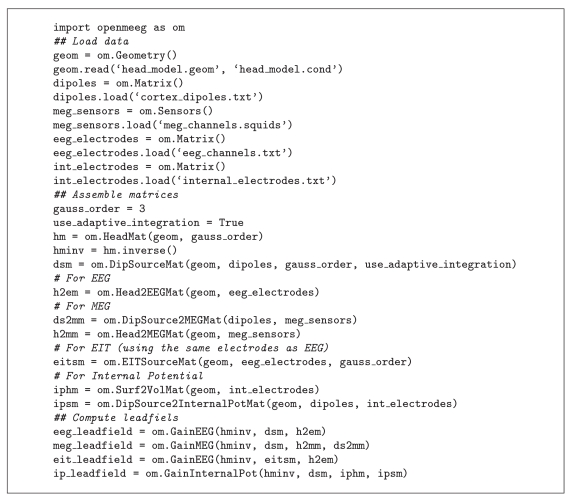
Demo script for computing the 4 types of lead fields with OpenMEEG in python.

**Algorithm 2:**
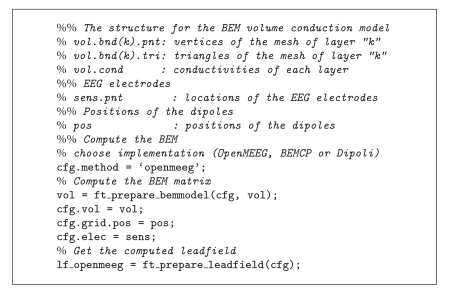
Demo script for computing an EEG lead field with OpenMEEG in FieldTrip.
